# Nef as a driver of immunodeficiency and HIV-associated diseases: insights from mouse models

**DOI:** 10.3389/fimmu.2026.1837301

**Published:** 2026-07-31

**Authors:** A.S. Yakovleva, E.A. Gorshkova, E.O. Gubernatorova, M.I. Bukrinsky, S.A. Nedospasov, M.S. Drutskaya

**Affiliations:** 1 Engelhardt Institute of Molecular Biology, Russian Academy of Sciences, Moscow, Russia; 2 Faculty of Biology and Belozersky Institute of Physico-Chemical Biology, Lomonosov Moscow State University, Moscow, Russia; 3 Department of Microbiology, Immunology and Tropical Medicine, The George Washington University School of Medicine and Health Sciences, Washington, DC, United States; 4 Sirius University of Science and Technology, Federal Territory Sirius, Krasnodarsky krai, Russia

**Keywords:** HIV-associated comorbidities, humanized mice, mouse models, Nef-induced inflammation, Nef-transgenic mice

## Abstract

Nef is a non-structural regulatory protein of human immunodeficiency virus (HIV) that exerts pleiotropic effects on the immune system. In infected cells, Nef modulates the surface expression of molecules critical for immune responses and regulates cytokine production, vesicular trafficking, and lipid metabolism as well as apoptosis and autophagy. Despite significant improvements in antiretroviral therapy (ART), Nef is detected in the circulation of HIV-infected individuals as a soluble protein and in association with virions and extracellular vesicles, and it is regarded as a driver of a broad spectrum of HIV-associated comorbidities. Serum Nef concentrations range from a few to several tens of ng/mL, comparable to systemic levels of certain proinflammatory cytokines in inflammatory diseases. This reflects the fact that the primary sources of Nef are cellular viral reservoirs in which ART does not suppress viral protein expression. Among these reservoirs, long-lived HIV-infected memory CD4^+^ T cells and tissue-resident macrophages are considered the major contributors to systemic Nef production. Due to the need for a more comprehensive understanding of the *in vivo* functions of Nef and the molecular mechanisms by which it contributes to HIV-associated pathogenesis, a variety of mouse models expressing Nef were developed. This review summarizes existing transgenic mouse models with cell type-specific Nef expression and humanized mouse models used in HIV research, including models based on Nef-deficient strains. The development and systematic characterization of such models are essential for providing experimental validation for the clinical implementation of Nef inhibitors in combination with established ART.

## Introduction

Human immunodeficiency virus (HIV) was described more than 40 years ago. Initial antiretroviral therapies capable of slowing disease progression were developed shortly thereafter, followed by the introduction of combination antiretroviral therapy (ART), which transformed HIV infection from a lethal disease to a manageable chronic condition. As a result, mortality associated with HIV infection has declined substantially. However, it remains higher than that observed in HIV-negative individuals (1). More than 40 million people worldwide are currently living with confirmed HIV infection, approximately 75% receive ART, yet more than 630,000 individuals still die each year from HIV-related causes (2). ART effectively reduces circulating viral loads to undetectable levels, enabling people living with HIV to maintain normal quality of life (3). Nevertheless, although individuals receiving ART are less likely to develop opportunistic infections, they remain susceptible to a wide range of HIV-associated comorbidities (4), including cardiovascular dysfunction (5, 6), renal impairment (7), pulmonary complications (8), and neurocognitive disorders (9, 10). HIV persistence is mediated by viral reservoirs that harbor HIV-infected memory CD4^+^ T cells, macrophages, monocytes, dendritic cells, and microglia (11). The development of HIV-associated pathologies in patients receiving ART is closely connected to the resistance of viral reservoir cells to therapy (12). Specifically, the anatomical localization of HIV-infected cells, including long-lived tissue-resident macrophages and microglia deeply embedded within the peripheral tissues and the brain, limits penetration of ART to clinically effective concentrations into relevant cellular compartments. Moreover, all clinically approved antiretroviral compounds prevent new rounds of infection, targeting discrete stages of HIV replication (attachment, reverse transcription, integration, and others) (13), but do not affect transcription of the already integrated provirus. As a result, within viral reservoirs that are relatively resistant to the cytopathic effects of HIV gene expression, viral transcription persists at low levels, producing viral proteins and occasionally virions, thus enabling long-term reservoir maintenance, production of pathogenic factors, and re-infection of new targets after discontinuation of ART (14).

Nef is a regulatory protein of both HIV-1 and HIV-2 that plays a central role in viral pathogenesis and in the development of HIV-associated comorbidities. The importance of Nef for the HIV infectious capacity and disease progression was highlighted in a cohort infected via blood transfusion with an HIV-1 strain carrying the deletion of the *nef* gene. Such individuals showed a markedly lower disease progression, milder clinical manifestations, and failed to develop Acquired Immunodeficiency Syndrome (AIDS) (15, 16). Importantly, in patients receiving ART, Nef can be produced from defective yet transcriptionally active proviruses (17) within viral reservoirs represented by memory CD4^+^ T cells (18) and tissue-resident macrophages (19, 20). Nef is detectable in circulation in multiple forms: associated with virions (21), as a soluble protein (22), as an intracellular protein within infected or exposed cells (23), and in extracellular vesicles (24). Notably, Nef is present in the blood of some HIV-infected patients on ART in concentrations reaching 8–11 ng/mL (22, 25). The amount of Nef secreted by cells within extracellular vesicles (exNef) correlates with the severity of chronic HIV infection (26).

Nef lacks intrinsic enzymatic activity. Instead, it acts by engaging host proteins and membrane-associated complexes. Accordingly, Nef exerts a broad functional repertoire, including downregulation of surface MHCI (27); MHCII (28); CD4 (29); CD28 (30); context-dependent regulation of apoptosis and autophagy (31, 32); modulation of cholesterol homeostasis (33–35); and alteration of cytokine production, including MIP-1α, MIP-1β, IL-2, and IL-10 (36–38). Beyond immune modulation, Nef was shown to colocalize with tissue-resident macrophages in tumors. Nef promotes the degradation of the tumor suppressor p53 (39) and increases the expression of induced cytidine deaminase (AID) and the oncogenic protein c-MYC (40). Dysregulation of AID and c-MYC expression is closely associated with the development of Burkitt’s lymphoma, which occurs with increased frequency in HIV-positive patients. In the presence of oncogenic viruses, Nef contributes to angiogenesis and tumor growth (41). Via its effects on cholesterol homeostasis and lipid rafts, Nef alters the epigenetic profile of myeloid cells, promoting inflammatory responses (42). Collectively, these activities support a role for Nef in sustaining chronic inflammation, including the activation of myeloid cells (43, 44). Persistent Nef expression and release from viral reservoirs likely represent key mechanistic links between ongoing immune dysregulation in patients undergoing ART and the pathogenesis of HIV-associated comorbidities.

**Figure 1:**
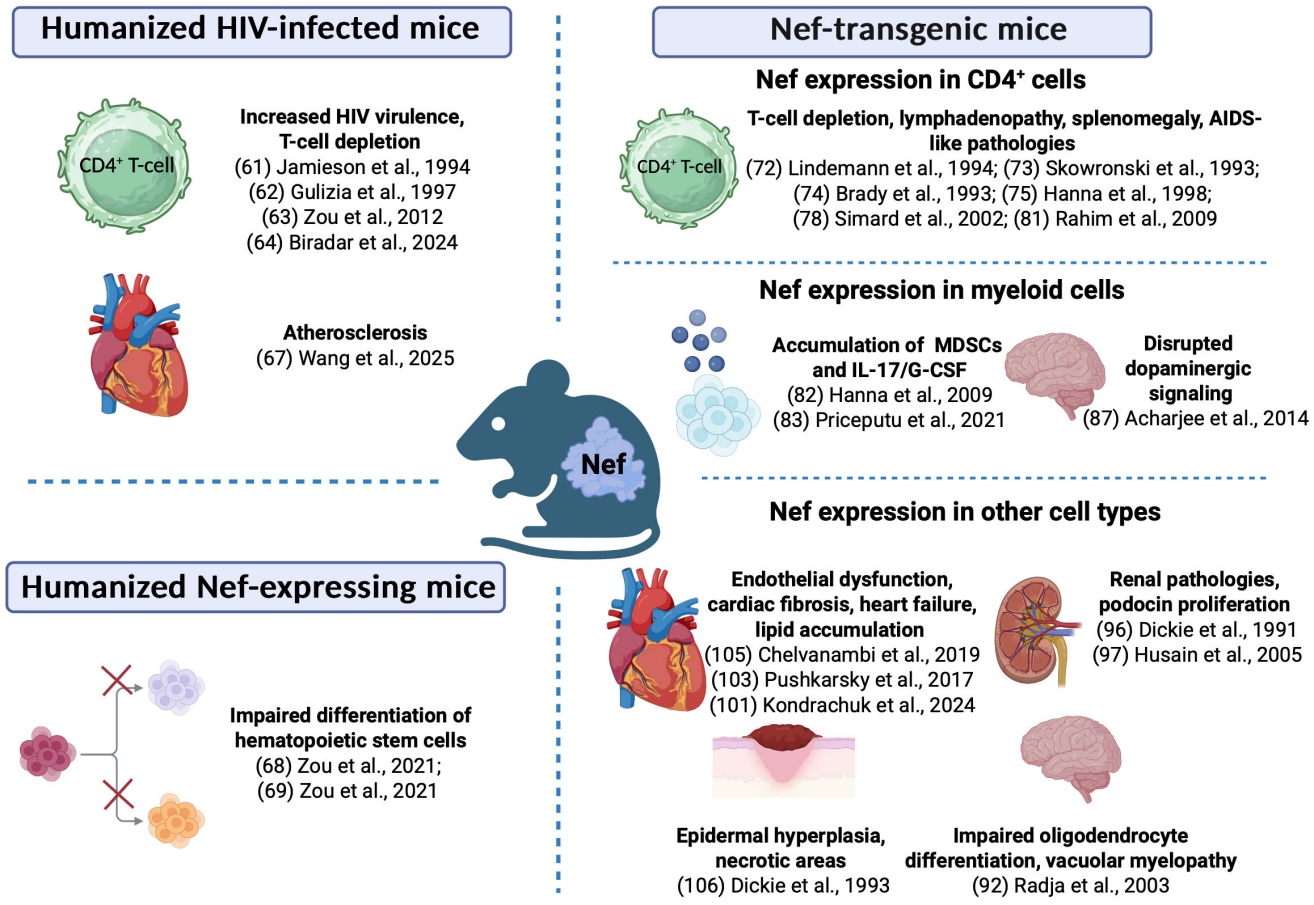
Mouse models allow to address Nef functions *in vivo*. Two different approaches were used to generate mouse models for studying Nef functions *in vivo*. The first involves humanized models of HIV infection in which Nef is expressed within its natural pathogenic context alongside other viral proteins. The second approach uses transgenic models that express Nef either constitutively or inducibly or in a tissue/cell type-specific manner. These allow investigation of Nef’s effects under homeostatic conditions or in combination with other experimental pathologies. The figure was created in BioRender (https://BioRender.com/y8gnj6v).

Since Nef plays an important role in HIV-associated comorbidities, there is an ongoing demand for the development of animal models that enable studies of its systemic and/or local tissue- and cell type-specific effects *in vivo* and provide accessible platforms for preclinical validation of Nef inhibitors (**Figure 1**). Several classes of molecules that are potential Nef inhibitors have been described (45). Some therapeutic strategies have focused on disrupting the ability of Nef to interact with cellular proteins by targeting specific amino acid motifs in Nef that mediate this interaction. One well-characterized example is the PxxP motif of Nef, which is responsible for binding and activation of Src family kinases (46). Low-molecular-weight inhibitors identified through structure-based screening and single-domain antibodies, including sdAb19, were shown to suppress this interaction, resulting in restoration of MHCI surface expression and reduction of viral replication (47–50). Other promising approaches involve the development of small molecules that induce Nef degradation or disrupt Nef dimerization. Such compounds could restore cell surface MHCI and CD4 expression and downregulate viral replication (51, 52). Inhibitors developed by Bukrinsky and colleagues target the ability of Nef to impair cholesterol transport by preventing Nef interaction with calnexin mediated by the lysines in the N-terminal region, thereby restoring ABCA1 function and cholesterol efflux in macrophages (53, 54). More recently, extracellular Nef and Nef-containing extracellular vesicles emerged as potential therapeutic targets. Finding that Nef is exposed on the surface of these vesicles opened up a possibility to target them with antibodies (55). However, these strategies remain at an early stage of development. It is still not clear whether systemic Nef inhibition will be sufficient to completely suppress Nef-associated pathologies, as inhibition of individual Nef structural motifs can prevent only certain effects (56). Another limitation that needs to be addressed is the localization of Nef-producing cells, including long-lived tissue-resident macrophages and microglia, deeply embedded in the peripheral tissues and in the brain, with limited penetration capacity for pharmacological substances.

The development of mouse models expressing Nef has made it possible to investigate the role of this protein in the pathogenesis of HIV-associated diseases *in vivo*. Humanized mouse models of HIV infection demonstrated that Nef is required for efficient viral replication and contributes to CD4^+^ T-cell depletion (**Figure 1**). Furthermore, studies using such mice revealed an important role for Nef in the development of HIV-associated atherosclerosis and impaired hematopoietic stem cell differentiation (**Figure 1**). At the same time, transgenic mouse models highlighted the substantial Nef contributions to a broad range of HIV-associated pathologies and provided valuable insights into the cellular and molecular mechanisms through which Nef affects diverse tissues and cell populations (**Figure 1**). Nevertheless, there remains a need to improve animal models of HIV infection in order to dissect the pathogenesis of Nef-associated diseases. This review discusses a broad spectrum of *in vivo* models that support mechanistic studies of Nef functions and allow evaluation of targeted therapeutics designed to inhibit its activity.

### Mouse models

Experimental modeling of HIV infection in animals aimed at defining the functions of individual viral proteins, tracking infection dynamics, and evaluating vaccines and therapeutic strategies were constrained by intrinsic resistance of mice and other commonly used laboratory species to HIV infection. Moreover, not all nonhuman primates are fully susceptible to HIV infection (57). Among available *in vivo* systems, chimpanzee *Pan troglodytes* (58) are considered the most relevant model because genetically they are closely related to humans and are susceptible to HIV-1. However, such studies are ethically questionable, technically challenging, time-consuming, and extremely expensive. Consequently, there remains an urgent need to develop genetically modified animal models that enable the investigation of HIV pathogenesis and HIV-associated diseases.

Over the past decades, the functions of Nef and its contribution to HIV pathogenesis were actively investigated. As noted above, Nef can affect multiple cellular populations, entering the cells not only through direct viral infection but also via its transport within extracellular vesicles (59). The effects and biological functions of extracellular vesicles cannot be fully reproduced *in vitro*, preventing comprehensive assessment of these aspects of Nef biology. To better characterize Nef-host interactions, a range of mouse models expressing the *nef* gene in various cell types have been developed, enabling the study of its functions *in vivo*. Collectively, these models provide deeper insights into the functions of Nef and allow evaluation of its impact on immune system regulation and other systemic or tissue-specific effects.

So far, two types of mouse models are employed to study Nef functions *in vivo*: transgenic models expressing *nef*, which allow the investigation of its effects under homeostatic conditions or in combination with other experimental pathologies, and humanized models of HIV infection, where Nef is expressed within its natural pathogenic context (**Figure 1**). Humanized models based on immunodeficient mouse strains such as SCID, NOD/SCID, or NSG are commonly classified by the strategy of human immune system reconstitution. These can include transplantation of human PBMCs, fetal tissues (thymus and liver), engraftment of CD34^+^ hematopoietic progenitor cells, or combined approaches that incorporate both tissue implants and hematopoietic cell transplantation. Transgenic mouse models, in contrast, are defined by the integration of the *nef* gene into the mouse genome under the control of promiscuous or tissue-specific regulatory elements, including humanized or inducible promoters. Genomic integration may occur randomly, as in conventional transgenesis, or be achieved through targeted approaches.

### Humanized mice

Early humanized mouse models capable of expressing and producing Nef were established using genetically engineered severe combined immunodeficiency (SCID) mice (60). These mice lack adaptive immunity due to the absence of functional B and T cells, while retaining an intact myeloid compartment. Lymphocyte populations were reconstituted by xenotransplantation of human tissue biopsies (61) or by engraftment of human peripheral blood cells, enabling subsequent HIV infection. Within this framework, Jamieson et al. humanized the immune system of SCID mice by implanting human fetal liver and thymus cells under the kidney capsule, generating SCID-hu Thy/Liv mice (62). SCID-hu Thy/Liv mice were infected with HIV strains containing deletions in various regulatory genes, including *nef*. These important experiments identified Nef as a key mediator of HIV pathogenesis and established its critical role in viral replication and the depletion of host T cells. The SCID-hu PBL model, in which the lymphocyte compartment of immunodeficient mice was reconstituted with human peripheral blood leukocytes prior to infection with various HIV isolates, demonstrated that the extent of Nef-mediated CD4^+^ Т cell depletion varies across viral strains (63).

The critical contribution of Nef to the depletion of CD4^+^ lymphocytes and double-positive thymocytes (CD4^+^CD8^+^) has been demonstrated in BLT humanized mouse models (64). In the classical BLT model NOD/SCID or NOD/SCID IL-2γ^−/−^ mice were implanted with human fetal thymus and liver tissue under the kidney capsule followed by infusion of autologous CD34^+^ hematopoietic progenitor cells. Infection of BLT mice with wild-type HIV-1 resulted in higher viral loads and more pronounced CD4^+^ T-cell depletion than infection with Nef-defective virus. Similar results have been reported using the BLTS model (65) in which NSG mice (Cg-Prkdc^scid^ Il2rg^tm1Wjl^/SzJ) received sublethal irradiation aimed at depleting myeloid cells prior to implantation of fetal thymus, liver, and spleen tissue. Subsequent reconstitution of hematopoiesis with human CD34^+^ cells followed by infection with wild-type HIV-1 or Nef-defective strains carrying deletions or mutations showed that functional Nef is required for full virulence and that it promotes immune dysregulation in both primary and secondary lymphoid organs, including the spleen (65). This study also demonstrated that Nef dimerization is critical for HIV replication, as mutations in the dimerization motif reduced viremia. Moreover, in the BLTS model, impairment of Nef dimerization reduced the surface expression of PD-1, a marker of T-cell exhaustion, thus increasing CD8^+^ T cell activity and supporting previous findings on Nef/p38-dependent inhibition of the antiviral response during HIV infection (66).

Concurrently, humanized models in which NSG mice were reconstituted exclusively with human CD34^+^ hematopoietic stem cells and then infected with wild-type HIV or a Nef-deleted virus gained popularity (67). Using such systems, Nef was shown to promote the development of atherosclerotic changes in HIV-infected humanized mice (68). The observed phenotype is associated with the ability of Nef-expressing cells to impair cholesterol efflux in macrophages by inhibition of the ABCA1 transporter, leading to their acquisition of a «foam cell phenotype» (35). In related NCG- and NSG-based models, mice were engrafted with CD34^+^ progenitor cells transduced with a Nef-expressing vector. This approach demonstrated that Nef disrupts hematopoietic stem/progenitor cell differentiation, impairing commitment to myeloid and erythroid lineages (69), as well as T-lymphocyte differentiation (70). However, the concept of productive infection of CD34^+^ cells by HIV remains controversial (71, 72). The Tsukamoto study questions the models generated by Zou et al. and suggests that these reported effects may be mediated by Nef-dependent premature death of progenitor cells or by inhibition of their differentiation. While hematopoietic progenitor cells are susceptible to HIV infection *in vitro*, the mechanisms underlying Nef involvement in hematopoiesis and hematopoietic stem cell differentiation *in vivo* remain an unresolved aspect of its immunobiology.

Collectively, the progressive refinement of humanized mouse models from SCID-hu to BLT/BLTS systems and CD34^+^ cell-based reconstitution not only confirmed the central role of Nef in viral virulence and suppression of immune responses but also improved understanding of its systemic effects (**Table 1**). A summary of humanized mouse models of HIV infection and Nef expression is provided in **Table 1**.

**Table 1:** Characterization of humanized mouse models for HIV infection and *nef* expression.

Humanized mice			
Mouse line	Generation	Reported effect of Nef	Reference
SCID-hu Thy/Liv	Transplantation of human fetal thymus and liver under the kidney capsule of SCID mice, followed by infection with HIV-1JR-CSF or HIV-1NL4-3 (with or without *nef* mutation).	Nef is required for efficient HIV-1 replication and cytopathic activity.	(62)
SCID-hu PBL	Transplantation of human peripheral blood leukocytes into SCID mice, followed by infection with HIV-1 strains SF2, R9, R9.BaL, or 89.6 (with or without *nef* deletion).	The impact of *nef* deletion varied depending on the viral isolate. Loss of Nef reduced CD4^+^ T cell depletion, diminished infection efficiency, and attenuated the cytopathic properties of the virus.	(63)
BLT	Immunodeficient NOD/SCID mice were implanted with human fetal thymus and liver, followed by reconstitution of hematopoiesis through infusion of CD34^+^ cells. The mice were subsequently infected with HIV-1 LAI (with or without *nef* deletion).	Nef is required for efficient viral replication and for the depletion of CD4^+^CD8^+^ thymic T cell precursors.	(64)
BLTS	Immunodeficient NSG mice were irradiated and then implanted under the kidney capsule with human fetal thymus, liver, and spleen, followed by intravenous infusion of CD34^+^ hematopoietic progenitor cells. Mice were subsequently infected with wild-type HIV, HIV NL4–3 carrying a nef deletion, or HIV-Y115D with disrupted Nef dimerization.	The absence of Nef reduced HIV virulence and attenuated HIV-mediated dysregulation not only in the blood and primary lymphoid organs, but also in secondary lymphoid tissues, including the spleen.	(65)
NSG-HSPCs	Immunodeficient NSG mice were engrafted with CD34^+^ hematopoietic progenitor cells and subsequently infected with wild-type HIV or the HIV-1 JR-CSF strain carrying a nef deletion.	Nef is a key driver of systemic inflammation and aortic changes that promoted the development of atherosclerosis in HIV-infected humanized mice.	(68)
NCG-HSPCs NSG-HSPCs	CD34^+^ hematopoietic cells were transduced with a vector expressing nef (HIV-1 LAI Nef). Immunodeficient NSG mice were irradiated and reconstituted through infusion of CD34^+^ cells with or without Nef expression.	Nef impaired the differentiation of hematopoietic stem cells into myeloid and erythroid lineages and T-lymphocytes.	(69, 70)

### Transgenic mice

To define cell type-specific mechanisms of Nef action and dissect its contribution to non-AIDS HIV pathology, transgenic mouse lines were developed in parallel with infectious humanized models to enable targeted Nef expression in defined cellular populations. However, these early studies overlooked the possibility that extracellular vesicles released from Nef-expressing cells could affect bystander cells. This mechanism may account for some observed effects in tissues lacking detectable Nef expression.

### Mice with Nef expression in CD4^+^ T cells

Early transgenic mouse models were designed to express Nef in CD4^+^ T cells, the primary targets of HIV during natural infection. Lindemann et al. generated mice carrying a transgenic *nef* gene under the control of the TCR β-chain promoter (HIV-1 NEF/3′LTR) (73). As expected, transgene expression was predominantly observed in the thymus and spleen. These mice developed pronounced lymphadenopathy and splenomegaly. Analysis of thymic cellular composition showed near-complete loss of CD4^+^ T-cell populations. Developmental profiling indicated that depletion occurred early during T-cell ontogeny. Notably, transplantation of bone marrow from wild-type donors restored T cell pools in the transgenic mice. Consistent with these changes, functional assessment revealed impaired T-cell responses indicating a T-cell defect attributable to Nef expression within this lineage.

Skowronski et al. generated transgenic mice expressing Nef from different viral isolates under the control of CD3 (74). In these models, transgene expression occurred predominantly in the thymus, resulting in impaired development of CD4^+^ T lymphocytes. Similar effects were reported for mice with transgenic Nef expression under control of human CD2 regulatory elements (LFA-2), a membrane protein involved in T lymphocyte intercellular adhesion (75). In addition to CD4^+^ T cell depletion, this study demonstrated reduced CD3-dependent activation of thymocytes.

Hanna et al. used the human CD4 promoter flanked by mouse CD4 enhancers to drive transgenic expression of whole HIV-1 coding sequences in target cells (76). In this system, all HIV-1 coding sequences were expressed in infection-relevant populations, including CD4^+^ T cells, CD4^+^CD8^+^ double-positive thymocytes, CD4^+^ macrophages and dendritic cells. The resulting CD4C/HIVWT Tg transgenic mice exhibited transgene expression in the thymus, spleen, and lymph nodes. Additionally, expression was detected in Kupffer cells, alveolar and peritoneal macrophages. Transgenic mice demonstrated reduced body weight, hypoactivity, diarrhea, and tremors. Most transgenic animals developed atrophy of the lymphoid tissue with near-complete loss of thymocytes and mature T cells, as well as pathological alterations in the kidneys and lungs. Subsequent studies demonstrated that Nef is the principal determinant of the observed AIDS-like syndrome (76), which was linked to the accumulation of immature dendritic cells and impaired antigen presentation (77). SH3-binding domain of Nef was shown to be responsible for these phenotypic manifestations (78). In a related CD4C/SHIV-*nefSIV* Tg transgenic model (79), expression of the *nef* gene derived from the simian immunodeficiency virus SIVmac239 was inserted under control of the human CD4C promoter. Despite differences in size and genomic organization between SIV and HIV Nef proteins, their similar functionality was demonstrated (80). Unlike HIV Nef, SIV Nef was unable to downregulate mouse CD4 surface expression (81). Yet, the overall phenotype of CD4C/SHIV-*nefSIV* Tg mice closely mirrored that of earlier CD4C-driven HIV Nef transgenic models. These findings suggest that the pathogenic effects of Nef in transgenic mice depend on multiple conserved signaling and immunomodulatory activities rather than solely on CD4 downregulation. A similar phenotype developed in adult mice with tetracycline-inducible Nef expression, suggesting that Nef predominantly impacts immune dysregulation during the postnatal rather than the embryonic period (82).

In summary, findings from CD4C/HIVWT Tg and CD4C/SHIV-*nef*SIV Tg models highlighted that even when transgene expression is limited to the CD4^+^ compartment, the pathological consequences of Nef expression extend beyond intrinsic T-cell defects. The observed phenotype of T-lymphocyte depletion in mice expressing Nef in CD4^+^ cells is likely associated with disturbances in T-cell homeostasis (**Table 2**). Collectively, these effects contribute to systemic reduction in the T-lymphocyte population. Presumably, T-cell extrinsic effects depend on intercellular interactions and lead to impaired antigen presentation and dysregulated humoral responses. These observations raise the question of the extent to which Nef can initiate or enhance pathology when expressed outside the T cell population, particularly in myeloid-lineage cells (monocytes/macrophages, dendritic cells), which serve as HIV reservoirs and represent regulators of inflammation. To address this, mice with Nef expression in myeloid cells were generated (**Table 2**).

**Table 2:** Transgenic mouse models with *nef* expression in distinct cell types.

Transgenic mice			
Name	Promoter, cell type	Phenotype	Reference
Mice with Nef expression in CD4^+^ T cells			
HIV-1 NEF/3’LTR	TCR β-chain promoter, Т-lymphocytes	CD4^+^ T-cell depletion, T-lymphocyte exhaustion, lymphadenopathy, splenomegaly	(73)
CD3 Nef1	CD3δ subunit promoter, Т-lymphocytes	Impaired development of CD4^+^ T lymphocytes	(74)
CD2 Nef	Regulatory elements of human CD2 (LFA-2), Т-lymphocytes	Impaired development of CD4^+^ T lymphocytes	(75)
CD4C/HIVWT Tg	Human CD4 promoter flanked by murine CD4 gene enhancers, whole HIV-1 coding sequences Т-lymphocytes	Absence of mature CD4^+^ T cells and CD4^+^CD8^+^ thymocytes, multiple organ pathologies, AIDS-like disease	(76)
CD4C/SHIV-*nefSIV* Tg	*nef* derived from SIVmac239 strain under control of human CD4 promoter, Т-lymphocytes	Splenomegaly, multiple organ pathologies, reduced numbers of mature CD4^+^ T cells and CD4^+^CD8^+^ thymocytes, impaired antibody isotype switching	(79)
TRE/HIVNef × CD4C/rtTA TRE/HIVNef × CD4C/rtTA2S-M2	Tetracycline-inducible nef expression under control of human CD4 promoter flanked by murine CD4 gene enhancers, Т-lymphocytes	CD4^+^ T-cell depletion, non-lymphoid pathologies, AIDS-like disease	(82)
Mice with Nef expression in myeloid cells			
CD68/HIVNef	CD68 promoter, Macrophages, dendritic cells	Absence of CD4^+^ T-cell depletion and non-lymphoid pathologies	(83)
CD11c/HIVNef	CD11c promoter, Dendritic cells	Absence of CD4^+^ T-cell depletion and non-lymphoid pathologies. Elevated IL-17 and G-CSF production, accumulation of MDSCs.	(83, 84)
c-fms/Nef	c-fms promoter, Macrophages, monocytes	Disrupted regulation of dopaminergic signaling, hyperactive behavior	(88)
Mice with Nef expression in other cell types			
MBP/HIV-1 Nef	MBP promoter, Oligodendrocytes	Impaired oligodendrocyte differentiation, vacuolar myelopathy	(93)
Tg26	Expression of all HIV-1 genes except the structural genes *gag* and *pol*	High mortality, renal pathologies characterized by proteinuria and progressive glomerulosclerosis, nephritis	(97)
Podocin/Nef	Cre-dependent under Podocin promoter Podocytes	Podocyte proliferation, loss of maturation markers	(98)
MyHC/Nef-TG	Cre-dependent under α-MyHC promoter Cardiomyocytes	Cardiac fibrosis, heart failure, premature mortality	(102)
PGK/Nef	PGK promoter Broad range of cells and tissues	Lipid accumulation in the aorta and liver	(104)
VE-cadherin/Nef	VE-cadherin promoter Endothelial cells	Endothelial dysfunction	(106)
MMTV/Nef	Nef expression in LTR MMTV–targeted organs Mammary gland, salivary glands, seminal vesicles, Harderian gland	None	(107)
HIV/Nef	Nef expression in LTR HIV–targeted organs Skin	Spontaneous epidermal hyperplasia with necrotic areas, basal keratinocyte overgrowth, alopecia areas	(107)

### Mice with Nef expression in myeloid cells

HIV can infect not only T lymphocytes but also CD4^+^ myeloid cells, including long-living tissue-resident macrophages, which act as natural viral reservoirs. In this regard, Hanna et al. generated a broad panel of transgenic mice expressing *nef* in distinct cell types to address the relationship between specific cellular sources of Nef and the corresponding phenotypic outcomes. This panel included mice with *nef* expression predominantly in macrophages and dendritic cells (CD68/HIVNef) and mice with expression restricted specifically to dendritic cells (CD11c/HIVNef) (83). Importantly, none of these models expressing *nef* exclusively in myeloid cells demonstrated CD4^+^ T lymphocyte depletion, suggesting that Nef production within T cells is necessary for lymphopenia. In the CD68/HIVNef line, Nef expression was detected mainly in peritoneal macrophages as well as spleen and lymph nodes, whereas in the CD11c/HIVNef line, its expression was observed selectively in spleen and lymph nodes. The absence of non-lymphoid pathologies in these mice, such as renal and pulmonary abnormalities reported in CD4C/HIVNef Tg, suggested that these manifestations are mediated by CD4^+^ cells outside the CD11c^+^ or CD68^+^ populations. At the same time, CD11c/HIVNef mice displayed elevated serum levels of IL-17 and G-CSF, which correlated with accumulation of CD11b^+^Gr1^+^ cells, representing a population of myeloid-derived suppressor cells (MDSCs) (84). The presence of immature or functionally defective dendritic cells (DCs) was demonstrated in mice expressing Nef and other HIV proteins in CD4^+^ cells (77), including DCs. Nef is known to activate the PAK2 kinase, promoting an immature DC phenotype and reducing antigen-presentation capacity (85). Functionally immature DCs, in turn, can enhance IL-17 production by CD4^+^ T cells (86). Elevated IL-17 levels stimulate G-CSF production, thereby promoting myelopoiesis and the accumulation of MDSCs (87).

Acharjee et al. developed a model of targeted Nef expression in the monocyte/macrophage lineage, including microglia, by placing *nef* under the control of the macrophage-specific *c-fms* promoter (88). In this model, transgene expression was detected in microglial cells. The authors reported that CCL2-dependent disruption of dopaminergic signaling was associated with hyperactive behavior in the transgenic mice. In humans, a Nef-mediated increase in CCL2 production was associated with a wide range of neurocognitive disorders affecting HIV-positive individuals (89). It was previously demonstrated that Nef can be transferred to endothelial cells, leading to their activation and enhanced CCL2 (MCP-1) production (90). In parallel, Nef activates the ERK signaling pathway and upregulates the adhesion molecule ICAM-1 on the endothelial cell surface (91). These changes contribute to blood-brain barrier dysfunction and may facilitate the entry of HIV-infected cells into the central nervous system. Furthermore, Nef binding to calmodulin in astrocytes modulates the MAPK signaling cascade, resulting in increased CCL2 production (92). Thus, Nef-mediated upregulation of CCL2 may contribute to neuroinflammation and behavioral abnormalities observed in HIV infection.

Collectively, these models demonstrated that myeloid-specific Nef expression can drive substantial proinflammatory and neuroimmune alterations, including induction/accumulation of MDSC-like populations and perturbation of neuroimmune regulation without causing CD4^+^ lymphopenia (**Table 2**). These findings underscore the strong cell type specificity of Nef pathogenic effects and highlight the importance of the cellular source of Nef when interpreting systemic manifestations of HIV infection.

### Mice with Nef expression in other cell types

A variety of transgenic mouse models were developed in which Nef is expressed in cell types that are not naturally susceptible to HIV infection. Although such systems are not physiologically relevant, they enable the investigation of the intrinsic effects of Nef on specific cell populations. Moreover, these models mimic the *in vivo* situation in which Nef is released within extracellular vesicles and subsequently taken up by neighboring cells that do not themselves produce the protein.

In a transgenic model in which Nef expression is driven by the myelin basic protein (MBP) promoter, oligodendrocytes represent the principal Nef-producing cell type (93). In this setting, Nef was shown to perturb oligodendrocyte differentiation, leading to the development of vacuolar myelopathy. Oligodendrocytes are not classical target cells for HIV, and whether they can be productively infected remains controversial. It is generally accepted that productive HIV replication in oligodendrocytes is minimal or absent (94). Nevertheless, HIV can influence oligodendrocyte function through viral proteins, including Nef. Recent studies demonstrated that Nef-containing extracellular vesicles mediate oligodendrocyte damage *in vivo*. Schenk et al. (95) confirmed findings previously reported by Radja et al. (93), demonstrating that intracranial injection of Nef EVs into mice caused demyelination and structural damage to oligodendrocytes in the central nervous system. Mechanistically, part of this damage was attributed to Nef-mediated inhibition of the ABCA1 cholesterol transporter, as myelin production by oligodendrocytes critically depends on ABCA1-mediated cholesterol efflux (96). Further studies are needed to elucidate the precise pathways by which Nef disrupts oligodendrocyte function.

The Tg26 transgenic mouse line, which expresses most HIV-1 genes but lacks the structural genes *gag* and *pol* (97), was widely used to study HIV-associated nephropathy and related renal diseases. Tg26 mice exhibit high mortality and severe kidney pathology characterized by proteinuria, progressive glomerulosclerosis, and nephritis. Based on these observations, Husain et al. developed a Cre-dependent transgenic mouse model with podocyte-specific expression of Nef under the *Podocin* promoter (98), demonstrating that Nef drives podocytes toward a proliferative phenotype accompanied by the loss of maturation markers such as WT1, synaptopodin, and Ki-67. Although renal epithelium is not considered a classical target of HIV infection due to the lack of CD4 and CXCR4/CCR5 expression, several studies suggest the presence of an HIV reservoir in renal epithelial cells (99, 100). Nef was shown to induce podocyte proliferation via activation of Src family kinases and STAT3 signaling pathways (101).

More recently, a Cre-dependent model enabling cardiomyocyte-restricted Nef expression under the *α-MyHC* promoter was generated (102). In this model, Nef induced fibrotic cardiac remodeling, leading to heart failure and premature mortality. The phenotype was proposed to be linked, at least in part, to Nef-mediated decline of autophagy. HIV does not efficiently infect cardiomyocytes; however, Nef has been detected in the cardiac tissue of people living with HIV (103). Recent studies showed that interaction of Nef with Beclin-1 and Rab7 interferes with the autophagic machinery, leading to impaired autophagic flux and increased cellular cytotoxicity (103). Kondrachuk et al. further demonstrated the accumulation of not only Beclin-1 but also Bcl-2, an additional negative regulator of autophagy in cardiomyocytes (102).

Another Cre-dependent transgenic mouse line expressing Nef together with an EGFP reporter was described (104). In that case, Nef expression was driven broadly across tissues under the control of the *PGK* promoter. Despite relatively low Nef levels, mice developed lipid accumulation in the liver and aorta, a phenotype that may contribute to cardiovascular pathology. Nef is known to impair cholesterol efflux from macrophages by inhibiting the ATP-binding cassette transporter ABCA1 (35) by disrupting its interaction with calnexin (105). In the model described by Pushkarsky et al., Nef was expressed broadly, and lipid droplet accumulation was observed in the liver but not in macrophages, suggesting that intracellular Nef can induce lipid accumulation in multiple cell types, extending beyond macrophage populations (104). One key aspect of Nef-induced cardiovascular pathology was established using transgenic mice expressing Nef under the control of the endothelial-specific VE-cadherin promoter (106). Endothelial-specific expression of Nef was sufficient to impair endothelium-dependent vasodilation of the aorta and to induce vascular remodeling. These findings establish a mechanism by which Nef persistence could contribute to ongoing HIV-related vascular dysfunction. Interestingly, HIV does not infect endothelial cells; therefore, the effects of Nef on endothelial cells are most likely attributable to circulating Nef EVs, as well as HIV-infected blood monocytes and perivascular macrophages—a hypothesis that could be addressed using previously generated myeloid-specific Nef-transgenic mice (**Table 2**).

In the model reported by Dickie et al. (107), *nef* expression was driven either by the HIV LTR or by the MMTV LTR. In MMTV/Nef mice, transgene expression was detected mainly in tissues typically targeted by MMTV, including the mammary gland, salivary glands, seminal vesicles, and Harderian gland. In contrast, in HIV/Nef mice, the primary site of Nef production was the skin. HIV/Nef transgenic mice developed spontaneous epidermal hyperplasia with focal necrotic lesions, consistent with increased proliferation of basal keratinocytes. Since the HIV-1 LTR can be activated by ultraviolet irradiation (108), these animals were also examined under UV exposure. UV-C irradiation increased Nef expression and was associated with further epidermal thickening. Keratinocytes, like other epithelial cells, are not susceptible to HIV infection. Nevertheless, Nef can penetrate certain epithelial cells, including those of the intestinal epithelium (109). The mechanisms by which Nef affects keratinocyte proliferation and skin homeostasis remain unclear and require further investigation.

Overall, tissue-specific transgenic models demonstrated that even in the absence of full viral replication, Nef can act as an autonomous driver of organ pathology (**Table 2**). Reported phenotypes range from impaired oligodendrocyte differentiation and vacuolar myelopathy to nephropathy, cardiac fibrosis, metabolic alterations in the liver, and proliferative skin lesions. Collectively, these observations (summarized in **Table 2**) underscore the broad spectrum of cell type-dependent effects mediated by Nef and support its potential contribution to non-immune complications of HIV infection.

Finally, a distinct mouse model of HIV infection is the EcoHIV system (110). Since HIV-1 cannot directly infect BALB/c mice due to species-specific restrictions, Potash et al. engineered a chimeric virus by replacing the HIV-1 gp120 gene with gp80 from the ecotropic murine leukemia virus. This virus was administered intravenously to mice, and infection efficiency was subsequently evaluated. Viral levels in the blood peaked at 2 weeks post-infection, and by 3 weeks, integrated virus was detected in lymphocytes, macrophages, and brain cells at levels comparable to those observed in human infection. However, viral replication rapidly declined after 2–3 weeks due to the host immune response, driving EcoHIV into a latent state resembling ART-suppressed HIV infection (111). Using this system, Schenck et al. compared infection with EcoHIV carrying or lacking the *nef* gene (112). Mice infected with Nef-deficient EcoHIV exhibited reduced expression of pro-inflammatory markers, highlighting a critical role for Nef in driving systemic inflammation. Furthermore, the study suggested that Nef contributes to myelin damage; however, this effect may be secondary to Nef-mediated microglial activation and chronic inflammation, and the precise mechanism underlying neuronal myelin damage remains to be elucidated.

## Conclusions

Phenotypes described in the mouse models of Nef expression partially recapitulate pathologies observed during HIV infection in patients, both on and off ART (**Tables 1**, **2**). A major limitation of transgenic mouse models, compared with natural HIV infection or HIV-infected humanized mice, is the constitutive and unregulated expression of a single viral protein within a defined cell type. In contrast, HIV-associated diseases develop in the context of simultaneous expression of multiple viral proteins affecting multiple bystander cells. Several HIV proteins besides Nef, including Tat, Vpr, and gp120, have been implicated in the pathogenesis of neurocognitive disorders (113, 114), endothelial dysfunction (115), kidney disease (116), chronic inflammation (117), and other HIV-associated comorbidities. The pathological manifestations observed in people living with HIV cannot be attributed to the action of a single viral protein, but rather result from the combined and often synergistic effects of multiple viral factors. Nevertheless, the present review specifically focuses on the systematic analysis of existing mouse models developed to elucidate the biological functions of HIV-1 Nef at the systemic, tissue-specific, and cell type-specific levels, as well as in the context of HIV infection with or without Nef expression in humanized mice (**Figure 1**). This focus is justified by the central role of Nef in HIV pathogenesis, its persistent expression despite suppressive ART, and extensive evidence implicating Nef in chronic inflammation, immune dysregulation, viral persistence, and non-AIDS comorbidities, even though other viral proteins may also contribute to these processes.

Clinical relevance and translational implications of Nef-specific mouse models derive from their ability to dissect key aspects of HIV pathogenesis that persist in people living with HIV (**Figure 1**). Despite effective viral suppression, patients on ART experience chronic inflammation, immune dysfunction, and a heightened risk of comorbidities, largely driven by persistent Nef production from viral reservoirs. Transgenic and humanized mouse models provided a mechanistic insight into how Nef alone induces CD4^+^ T-cell depletion (73–76), myeloid cell activation, foam cell formation (35, 104), and tissue-specific pathologies such as endothelial cell-dependent impaired vasodilation in the aorta (68, 106), cardiac fibrosis (102), podocyte proliferation (98), and oligodendrocyte damage (93) (**Tables 1**, **2**). These findings directly parallel clinical observations, where circulating Nef correlates with inflammatory markers and disease severity (26). Most importantly, these models provide a platform for validating Nef-targeted inhibitors *in vivo*, which are not currently part of standard ART protocols (45). Additionally, by dissecting cell type-specific functions of Nef, these models may facilitate the development of adjunctive therapies aimed at reducing persistent inflammation and preventing organ-specific damage. Ultimately, mouse models of Nef expression help to bridge the gap between mechanistic *in vitro* studies and clinical application, offering a rationale for adding Nef inhibitors to ART to improve long-term outcomes of therapy.

Although *in vitro* approaches are invaluable for dissecting intrinsic Nef functions and regulatory mechanisms, their translational relevance is limited, as they do not address the context of the living organism. Therefore, mouse models discussed here provide a powerful tool for studying Nef functions, including their ability to reproduce the response to HIV infection in humanized immunodeficient mice reconstituted with the components of the human immune system. At the same time, several limitations must be considered when interpreting data from such models. First, although humanized mouse models reproduce key elements of the human immune system, experimental outcomes depend strongly on the efficiency, lineage composition, and inter-animal variability of human cell engraftment. In particular, incomplete or inconsistent reconstitution of myeloid populations, tissue-resident macrophages, and microglia, together with species-specific differences in tissue microenvironments and cytokine networks, can substantially influence HIV infection dynamics, immune activation, and inflammatory responses. Second, transgenic lines with tissue- or cell-specific *nef* expression often exhibit non-physiological expression levels, lack other viral proteins and the replication cycle, and may be affected by insertion-site artifacts. Moreover, pathologies caused by targeted Nef expression (for example, in cardiomyocytes and oligodendrocytes), although informative for defining possible Nef-dependent cellular programs, are not fully physiological since these cells cannot be infected by HIV and therefore do not produce Nef during natural infection. Third, humanization does not fully restore the immune functions of immunodeficient mice, which, together with graft-versus-host disease, limits their utility as true models of HIV infection. Finally, interspecies differences in Nef molecular targets and downstream signaling pathways can lead to underestimation of human-relevant interactions or, conversely, to mouse-specific artifacts. Further research combining cell type-specific transgenic Nef expression with humanized mouse models and infection with Nef-deficient HIV, although complex and expensive, should allow the separation of the effects caused by viral replication from those directly related to Nef expression in specific cell populations. The use of improved humanized mice with a functional human immune system and suppressed graft-versus-host responses should provide a more physiological model of HIV infection *in vivo* and enable a detailed investigation of Nef’s contribution to the development of immune dysfunction, chronic inflammation, and HIV-associated diseases.

Nef-expressing transgenic mice are particularly useful for identifying the specific cellular sources and tissue compartments that contribute to the development of HIV-associated pathologies. Such insights can lead to targeted interventions aimed at preventing Nef-mediated cellular dysfunction. Importantly, the development and validation of Nef inhibitors remains a key goal because anti-Nef agents are not currently part of standard ART. Several Nef-binding molecules have been reported to partially attenuate Nef pathogenic effects (47, 49, 118, 119), but inhibiting individual interaction sites typically yields only partial suppression of overall Nef activity, so search for a more universal Nef inhibitor is warranted. The development and implementation of anti-Nef therapeutics could significantly improve current treatment strategies and long-term outcomes for people living with HIV by limiting progression of multiple HIV-associated comorbidities.
